# AMP-Activated Protein Kinase Attenuates High Salt-Induced Activation of Epithelial Sodium Channels (ENaC) in Human Umbilical Vein Endothelial Cells

**DOI:** 10.1155/2016/1531392

**Published:** 2016-08-22

**Authors:** Wei-Wan Zheng, Xin-Yuan Li, Hui-Bin Liu, Zi-Rui Wang, Qing-Qing Hu, Yu-Xia Li, Bin-Lin Song, Jie Lou, Qiu-Shi Wang, He-Ping Ma, Zhi-Ren Zhang

**Affiliations:** ^1^Departments of Cardiology and Clinical Pharmacy, Harbin Medical University Cancer Hospital, Institute of Metabolic Disease, Heilongjiang Academy of Medical Science, Key Laboratories of Education Ministry for Myocardial Ischemia Mechanism and Treatment, Harbin 150000, China; ^2^Department of Clinical Pharmacy, Institute of Clinical Pharmacy, The 2nd Affiliated Hospital, Harbin Medical University, Harbin 150086, China; ^3^Department of Physiology, Emory University School of Medicine, Atlanta, GA 150040, USA

## Abstract

Recent studies suggest that the epithelial sodium channel (ENaC) is expressed in the endothelial cells. To test whether high salt affects the NO production via regulation of endothelial ENaC, human umbilical vein endothelial cells (HUVECs) were incubated in solutions containing either normal or high sodium (additional 20 mM NaCl). Our data showed that high sodium treatment significantly increased *α*-, *β*-, and *γ*-ENaC expression levels in HUVECs. Using the cell-attached patch-clamp technique, we demonstrated that high sodium treatment significantly increased ENaC open probability (*P*_*O*_). Moreover, nitric oxide synthase (eNOS) phosphorylation (Ser 1177) levels and NO production were significantly decreased by high sodium in HUVECs; the effects of high sodium on eNOS phosphorylation and NO production were inhibited by a specific ENaC blocker, amiloride. Our results showed that high sodium decreased AMP-activated kinase (AMPK) phosphorylation in endothelial cells. On the other hand, metformin, an AMPK activator, prevented high sodium-induced upregulation of ENaC expression and *P*_*O*_. Moreover, metformin prevented high salt-induced decrease in NO production and eNOS phosphorylation. These results suggest that high sodium stimulates ENaC activation by negatively modulating AMPK activity, thereby leading to reduction in eNOS activity and NO production in endothelial cells.

## 1. Introduction

Previous investigations have shown that dietary high salt intake impairs relaxation of blood vessels in response to vasodilator stimuli [1, 2]. A possible contributor to this impairment of vasodilator-stimulated vascular relaxation in animals on a high salt diet could be an impaired function of the endothelium, which normally plays a critical role in regulating vascular tone by generating vasodilator and vasoconstrictor chemicals [2, 3]. Nitric oxide (NO) is an important endothelium-derived relaxation factor, which is produced by the action of endothelial nitric oxide synthase (eNOS). Reduced NO release impairs the vasodilation of blood vessels, which promotes endothelial dysfunction [4]. A moderate increase in sodium concentration has been shown to stiffen cultured endothelial cells within minutes, thereby reducing NO release [5]. However, the mechanism by which changes in sodium concentration induce these cellular responses in the endothelium is currently unknown.

Epithelial sodium channel (ENaC) mediates sodium transport across the apical membrane of epithelia and is considered the predominant site for regulating sodium reabsorption in kidney, lung, colon, and sweat glands [6]. ENaC consists of three different subunits (*α*, *β*, and *γ*) that are expressed in a tissue specific manner [7, 8] and can be blocked by amiloride [9]. The regulation of ENaC is tissue specific and mediated by the mineralocorticoid hormone aldosterone and aldosterone-induced proteins, for example, the serum- and glucocorticoid-regulated kinase 1 [10, 11]. Thus, various proteins and extracellular factors interact directly or indirectly with ENaC [9, 12]. ENaC is the typical sodium channel found in a variety of epithelial cells of kidney, colon, and lung. However, recent studies indicate that ENaC is also expressed in vascular endothelial cell, where its roles are similar to that in the epithelia [13–15]. In endothelial cells, an acute application of aldosterone leads to amiloride-sensitive cell swelling and a reduction in NO release, which is probably caused by sodium and water uptake mediated by the endothelial ENaC [16]. Moreover, inhibiting endothelial ENaC activates eNOS and increases NO production in mesenteric arteries [17]. However, the role of ENaC in high salt-induced endothelium dysfunction is unknown.

The metabolic sensor AMP-activated kinase (AMPK) is ubiquitous metabolite-sensing Ser/Thr kinase that is a heterotrimer comprising catalytic *α*-subunit and regulatory *β*- and *γ*-subunits. AMPK activity increases under the conditions of metabolic stress in response to elevated intracellular AMP : ATP ratios [18]. It has been demonstrated* in vitro* that stimulation of AMPK by metformin, phenformin, and 5-aminoimidazole-4-carboxamide-1-beta-D-ribofuranoside (AICAR) inhibits ENaC conductance in* Xenopus* oocytes, HEK293 cells, and polarized renal epithelial cells [19, 20].

In this study, we aim to test whether high sodium treatment may affect eNOS activity and NO production levels by altering the expression profile and activity of ENaC via AMPK-dependent signaling.

## 2. Materials and Methods

### 2.1. Endothelial Cell Culture

HUVECs were cultured in endothelial cell growth medium (Hyclone, Logan, UT, USA) supplemented with 10% fetal bovine serum (Hyclone, Logan, UT, USA) plus 1% penicillin/streptomycin (Invitrogen, Carlsbad, CA, USA). Confluent cells were used for experiments between passages 3 and 6. When HUVECs had grown to 85–90% confluence in 6-well plates, they were incubated with or without different concentrations (10, 20, and 30 mM) of additional NaCl and maintained in 95% air and 5% CO_2_ at 37°C for 24 h.

### 2.2. Cell Viability Assay

Cell viability was estimated by measuring mitochondrial dehydrogenase activity, using the colorimetric MTT assay, based on the fact that viable cells (but not dead cells) can reduce 3-(4,5-dimethylthiazol-2-yl)-2,5-diphenyl tetrazolium bromide (MTT), as previously described in our previous works [21]. Briefly, cells were cultured in 96-well plates and treated with either 10, 20, or 30 mM NaCl in RPMI 1640 medium supplemented with 10% FBS for 24 or 48 h. The cells were then incubated with MTT solution (5 mg/mL) for 4 h. The formazan crystals, thus, formed were dissolved in dimethyl sulfoxide (DMSO) (150 *μ*L/well). The absorbance was recorded at a wavelength of 490 nm using a microplate reader (Tecan, Switzerland). All experiments were performed at least 3 times.

### 2.3. Patch-Clamp Studies

ENaC single-channel currents were recorded using cell-attached patch-clamp configuration using an Axon Multiclamp 200B amplifier (Axon Instruments, Foster City, CA, USA) at room temperature (22–25°C). HUVECs were thoroughly washed with a NaCl solution containing (in mM) 115 NaCl, 4.5 KCl, 1 MgCl_2_, 1 CaCl_2_, 5 HEPES, and 5 Na-HEPES, adjusted to pH 7.2 with NaOH. This NaCl solution was used as the bath solution for recordings. Patch pipettes were pulled from borosilicate glass with a Sutter P-97 horizontal puller, and resistance of the pipettes was ranged between 6 and 10 MΩ when filled with the NaCl solution. The data were acquired by application of 0 mV pipette potential and were sampled at 5 kHz and low-pass filtered at 1 kHz with Clampex 10.2 Software (Molecular Devices, Sunnyvale, CA, USA). Prior to analysis, the single-channel traces were further filtered at 30 Hz. ENaC activity was recorded for 2 min after the formation of the cell-attached mode and stabilization of ENaC activity. A single patch was typically recorded for at least 30 min and *P*_*O*_ was analyzed using at least 30 min recordings. The open probability (*P*_*O*_) of ENaC was calculated as follows: *P*_*O*_ = *NP*_*O*_/*N*, where *N* (*N* was estimated by the current amplitude histogram) represents the apparent number of active channels in the patch.

### 2.4. Western Blot Analysis for ENaC, eNOS, and AMPK

For western blot analysis, protein samples were extracted from HUVECs, separated by 10% SDS-PAGE, and transferred to nitrocellulose membrane using a Trans-Blot unit for 1.5 h at 250 mA. Membranes were blocked with 5% (wt/vol) nonfat milk in TBS (pH 7.4) containing 0.1% (vol/vol) Tween 20 (TBS-T) for 1 h at room temperature (25°C). Then, the membranes were incubated with primary antibodies against *α*-ENaC (StressMarq, Victoria, BC, Canada), phospho-eNOS (Ser1177; Thermoscientific, Waltham, MA, USA), eNOS (Abcam, NJ, USA), AMPK*α* and phospho-AMPK*α* (Cell Signaling Technology, Boston, MA, USA), and *β*-actin (Santa Cruz Biotechnology, USA) overnight at 4°C, followed by washing in TBS-T and incubation with the corresponding secondary antibodies (1 : 10,000) for another 1 h at 22–25°C. Membranes were finally washed with TBS-T and the protein bands were detected by ECL kit (Invitrogen, Carlsbad, CA, USA) and scanned densitometry (Bio-Rad, CA,USA).

### 2.5. Measurement of NO Production by Laser Confocal Fluorescence Microscopy

Fluorimetric measurements were performed on HUVECs using the Olympus Fluoview FV1000 laser scanning confocal system. 4-Amino-5-methylamino-2′,7′-difluorofluorescein diacetate (DAF-FM DA; Life Technology, Rockford, IL, USA) was used as the NO indicator. Briefly, DAF-FM DA (10 *μ*M) was added to the HUVECs for 1 h. Next, the labeled cells were washed twice in modified PBS before analysis using confocal microscopy. The amount of NO in response to high salt incubation was evaluated by measuring the fluorescence intensity at 515 nm upon excitation at 495 nm.

### 2.6. Statistical Analysis

All data are represented as mean ± SEM. Statistical analysis was performed using SigmaPlot and SigmaStat Software (Jandel Scientific, CA, USA). One-way ANOVA, ANOVA for repeated measurements (followed by Student-Newman-Keuls* post hoc* test), or Student's *t*-test was used for statistical analysis. Differences were considered statistically significant for *p* < 0.05.

## 3. Results

### 3.1. High Salt Treatment Increases ENaC Expression in HUVECs

**Figure 1:**
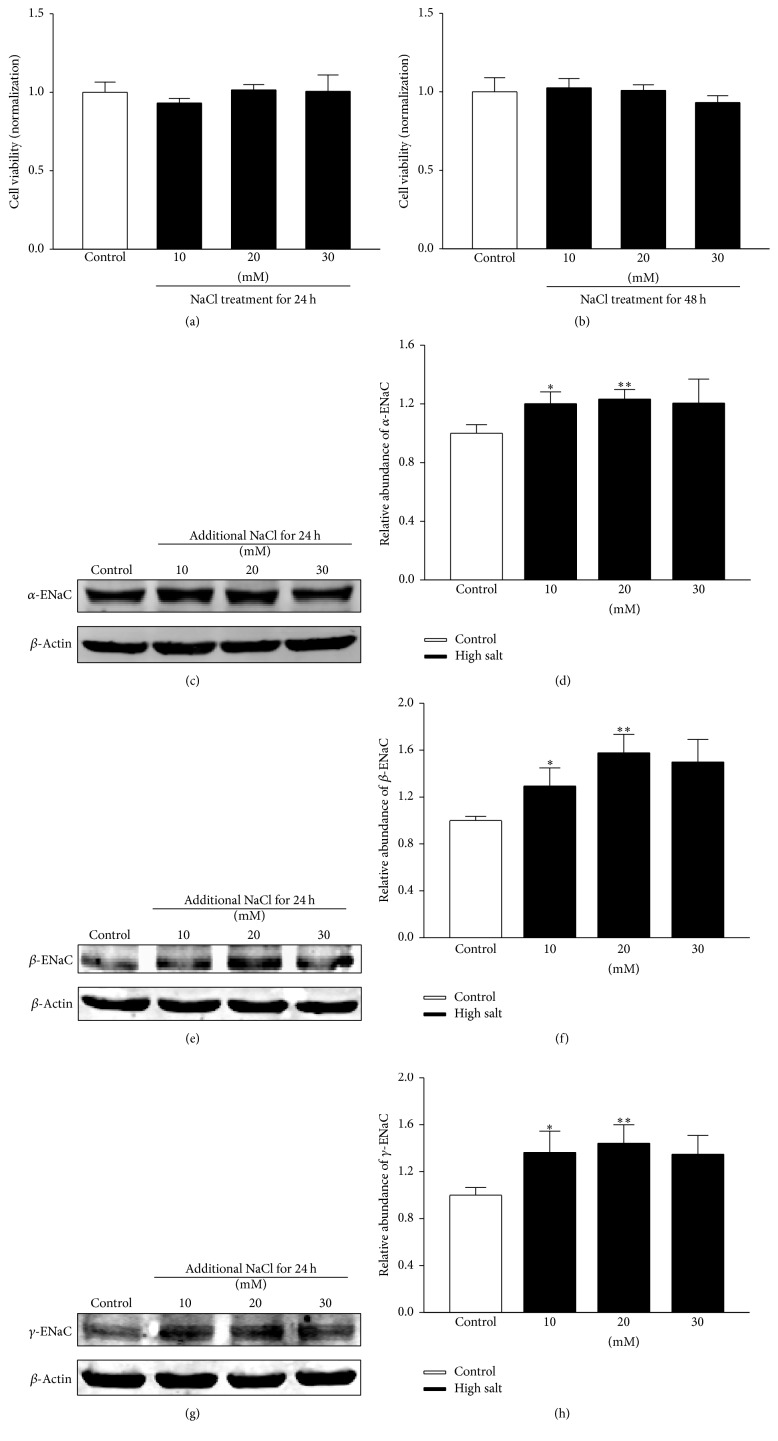
Effect of high sodium treatment on ENaC expression in HUVECs. ((a) and (b)) MTT assay was performed to measure the effect of high sodium concentration on cell viability. NaCl at concentrations of 10, 20, and 30 mM was, respectively, applied to the cells for (a) 24 h and (b) 48 h (*n* = 6 in each group). ((c)–(h)) Effects of high sodium application (additional 10, 20, and 30 mM NaCl treated for 24 h) on *α*-, *β*-, and *γ*-subunit levels of ENaC in HUVECs. Levels of ENaC subunits and *β*-actin were evaluated using western blot analysis. The densitometry values were normalized to *β*-actin (*n* = 5 in each group). *∗* indicates *p* < 0.05; *∗∗* represents *p* < 0.01 versus control.

We first determined whether incubation of HUVECs with high sodium could affect cell viability. Our data showed that treatment of the HUVECs with additional 10, 20, and 30 mM NaCl for up to 48 h did not affect cell viability (Figures 1(a) and 1(b)).

Our data show that *α*-, *β*-, and *γ*-subunits of ENaC are expressed in HUVECs. To test whether high salt alters the expression profile of ENaC, we incubated HUVECs with an aldosterone-free medium containing additional 10, 20, or 30 mM NaCl for 24 h. Our data show that addition of 10 mM and 20 mM NaCl but not 30 mM NaCl significantly enhanced the abundance of all the three subunits of ENaC compared with that in the normal-sodium condition (Figures 1(c)–1(h)). We then examined whether the effect of high salt on ENaC expression of HUVECs was due to the change in osmolarity. As the osmolarity of 20 mM of mannitol equals the osmolarity of 10 mM of NaCl, 20, 40, and 60 mM mannitol were, respectively, used to examine whether osmolarity can alter ENaC expression. We found that mannitol did not affect ENaC expression at any concentration we used (data not shown).

### 3.2. ENaC Activity in HUVECs Was Increased by High Salt

**Figure 2:**
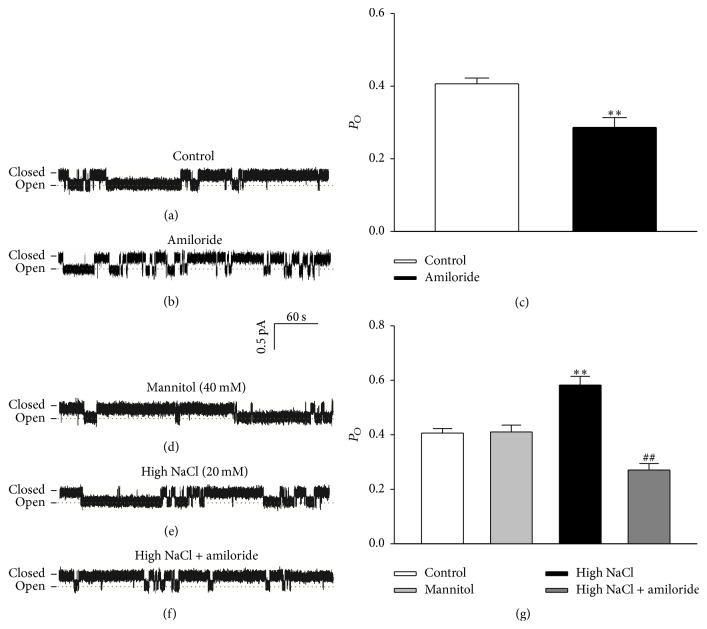
Effect of high sodium on ENaC activity in HUVECs. ((a) and (b)) Representative traces of ENaC single-channel current recorded from HUVECs with and without amiloride treatment. (c) Summarized *P*_*O*_ obtained from the single-channel recordings as shown in (a) and (b). The data show that *P*_*O*_ calculated from amiloride group significantly decreased compared to that from control group (*n* = 5; *∗∗* indicates *p* < 0.01 versus control group). ((d)–(f)) Representative traces of ENaC single-channel current recorded from HUVECs under indicated conditions. (g) Summarized *P*_*O*_ obtained from the single-channel recordings as shown in (d)–(f). The data show that *P*_*O*_ calculated from high NaCl group significantly increased compared to that from control group (*n* = 5 in each group). *∗∗* indicates *p* < 0.01 versus control group; ## represents *p* < 0.01 versus high NaCl group.

Since additional 20 mM NaCl treatment had the most impact on the expression levels of all three subunits of ENaC, we therefore chose additional 20 mM NaCl to treat HUVECs for 24 h followed by cell-attached patch-clamp analysis. Under control conditions, we detected a single-channel current with small amplitude in HUVECs (Figure 2(a)). This current was blocked by 0.5 *μ*M amiloride (Figure 2(b)). Furthermore, this amiloride-sensitive current was significantly upregulated by additional 20 mM NaCl, but not by additional 40 mM mannitol (Figures 2(c) and 2(d)). The high sodium-induced activation of amiloride-sensitive currents was significantly blocked by 0.5 *μ*M amiloride (Figure 2(e)). These results together suggest that the regulatory effect of high sodium on ENaC activity in HUVECs was not due to osmotic stress (Figure 2(f)).

### 3.3. ENaC Activity Contributes to High Salt-Induced Downregulation of eNOS Phosphorylation and NO Production

**Figure 3:**
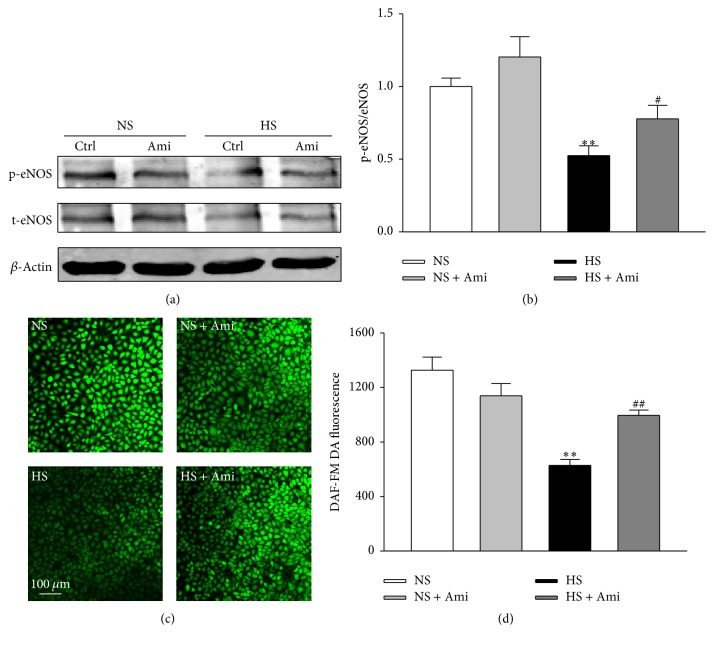
Effect of ENaC blockade on the phosphorylation of eNOS Ser 1177 and production of NO. (a) Representative western blots demonstrating the expression levels of total eNOS, p-eNOS, and *β*-actin in HUVECs cultured with normal sodium (NS), NS plus 0.5 *μ*M of amiloride (Ami), high sodium (additional 20 mM NaCl treated for 24 h; HS), and HS plus 0.5 *μ*M of amiloride (Ami). (b) Summaries of eNOS activity in response to amiloride in NS or HS group. (c) The images represent the levels of intracellular NO detected using membrane-permeable fluorescent probe, DAF-FM DA, under indicated conditions in HUVECs. (d) Summary of fluorescence results from (c) (*n* = 5 in each group). *∗∗* indicates *p* < 0.01 versus NS group; # represents *p* < 0.05; ## indicates *p* < 0.01 versus HS group.

Although accumulated evidence suggests that excess salt can stiffen the vascular endothelium and reduce NO release, it is presently not known whether the endothelial ENaC is involved in high sodium stimulated cellular responses. Western blotting experiments with total protein homogenates obtained from HUVECs showed that treatment with high sodium (additional 20 mM NaCl) for 24 h significantly reduced eNOS phospho-Ser 1177 levels. In contrast, ENaC blockade after using treatment with 0.5 *μ*M amiloride for 1 h significantly prevented high sodium-induced downregulation of eNOS phospho- Ser 1177 (Figures 3(a) and 3(b)).

To determine whether high sodium can decrease NO production, HUVECs were loaded with a NO-sensitive probe, DAF-FM DA. The fluorescent intensity was significantly reduced after addition of 20 mM NaCl, suggesting a reduction of NO production under this condition. Interestingly, the inhibitory effect of high salt on NO production was significantly restored by amiloride in HUVECs (Figures 3(c) and 3(d)). These results support the notion that ENaC activity may contribute to high sodium treatment-induced reduction of eNOS activity and NO production.

### 3.4. AMPK Attenuates High Salt-Induced Increase in ENaC Expression

**Figure 4:**
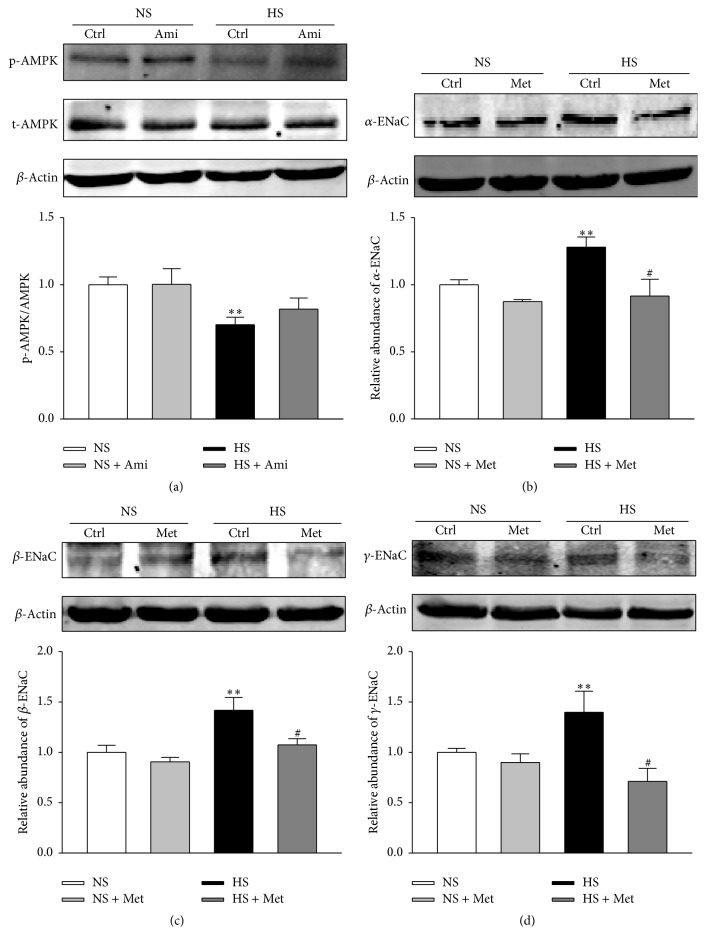
High salt-induced increase in expression of ENaC protein was attenuated by metformin (AMPK activator). (a) Representative western blots demonstrating the expression levels of total AMPK, p-AMPK, and *β*-actin in HUVECs cultured with normal sodium (NS), NS plus 0.5 *μ*M of amiloride (Ami), high sodium (additional 20 mM NaCl treated for 24 h; HS), and HS plus 0.5 *μ*M of amiloride (Ami). ((b)–(d)) Representative western blots demonstrating the expression levels of *α*-, *β*-, and *γ*- ENaC subunits in HUVECs cultured with NS, NS plus 2 mM metformin (Met), HS, and HS plus 2 mM metformin (Met) for 24 h. The densitometry values were normalized to *β*-actin (*n* = 5 in each group). *∗∗* indicates *p* < 0.01 versus NS group; # represents *p* < 0.05 versus HS group.

It has been demonstrated* in vitro* that AMPK inhibits ENaC [19, 20, 22]. Therefore, we reasoned that manipulation of AMPK activity may affect ENaC expression profile and/or ENaC activity. The data shown in Figure 4(a) suggest that AMPK activity was blunted by high sodium (Figure 4(a)). However, blocking ENaC by amiloride had no obvious effects on AMPK activity (Figure 4(a)). We speculated that the inhibition of AMPK activity might be a reason for enhancement of ENaC expression and activity. We then examined whether metformin, an AMPK activator, could reverse the high sodium treatment-induced increase in ENaC expression. Consistent with the results described above, the expression levels of *α*-, *β*-, and *γ*-ENaC were significantly upregulated by high sodium; however, the effect of high sodium on ENaC expression in HUVECs was almost completely diminished in HUVECs treated with 2 mM metformin for 24 h (Figures 4(b), 4(c), and 4(d)).

### 3.5. AMPK Activation Reduces High Salt-Induced Elevation of ENaC Activity

**Figure 5:**
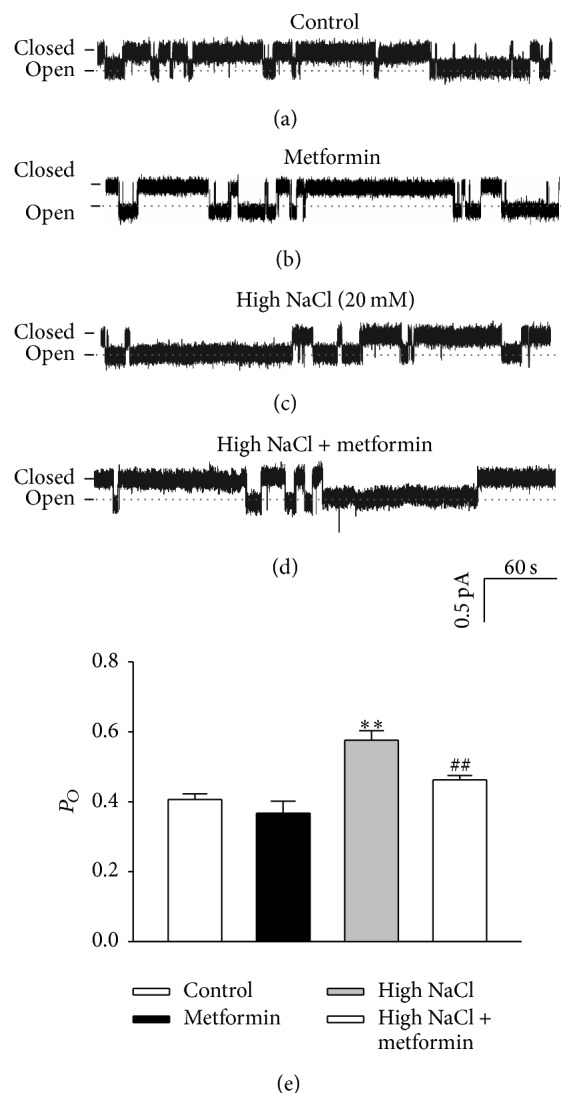
High salt-induced increase in ENaC activity was blunted by metformin. ((a)–(d)) Representative traces of ENaC single-channel current recorded from HUVECs under indicated experimental conditions. (e) Summarized *P*_*O*_ obtained from the single-channel recordings as shown in ((a)–(d)). The data showed that metformin significantly reduced ENaC *P*_*O*_ compared to that from high NaCl group (*n* = 5 in each group). *∗∗* indicates *p* < 0.01 versus control group; ## represents *p* < 0.01 versus high NaCl group.

We next determined whether AMPK activity contributes to the regulatory effect of high salt on ENaC *P*_*O*_. The data show that application of 2 mM metformin to HUVECs under control conditions had no effect on ENaC *P*_*O*_ (0.41 ± 0.02 to 0.37 ± 0.03), suggesting that metformin does not affect ENaC activity under control conditions (Figures 5(a), 5(b), and 5(e)). However, high sodium-induced increase in ENaC activity was significantly attenuated by application of 2 mM metformin (Figures 5(c), 5(d), and 5(e)). These results suggest that metformin exerts a protective effect on high sodium-induced enhancement of ENaC activity in HUVECs.

### 3.6. AMPK Activation Prevents High Salt-Induced Downregulation of eNOS Phosphorylation and NO Production

**Figure 6:**
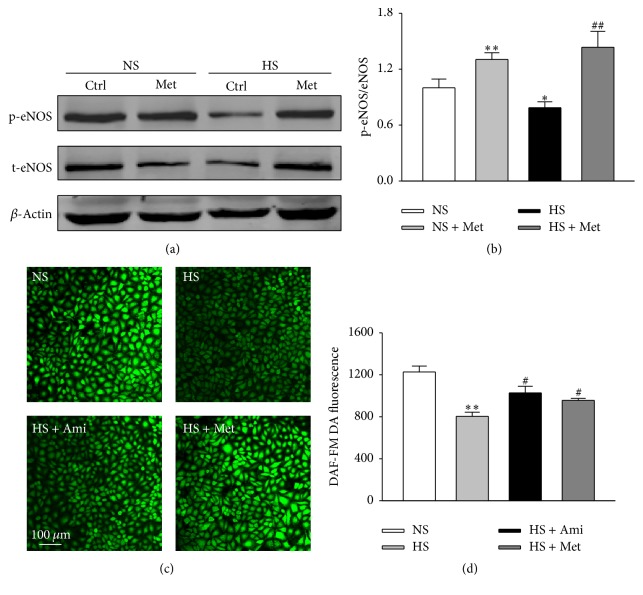
High salt-induced reductions in the levels of eNOS phosphorylation and NO production were partially reversed by metformin. (a) Representative western blots demonstrating the expression levels of total eNOS, p-eNOS, and *β*-actin in HUVECs cultured with NS, NS plus 2 mM metformin (Met), HS, and HS plus 2 mM metformin (Met) for 24 h. (b) Summaries of eNOS activity in response to metformin in NS or HS group. (c) The images represent the levels of intracellular NO detected by a membrane-permeable fluorescent probe, DAF-FM DA, under indicated conditions in HUVECs. (d) Summary of fluorescent intensity analyzed from the experiments shown in (c) (*n* = 5 in each group). *∗* and *∗∗*, respectively, indicate *p* < 0.05 and *p* < 0.01 versus NS group; # and ##, respectively, represent *p* < 0.05 and *p* < 0.01 versus HS group.

High salt concentration stimulates ENaC and leads to the reduction of eNOS activity and NO production. The stimulated ENaC expression and activity could be inhibited by an AMPK activator, metformin. Therefore, we tested whether AMPK activation could prevent high salt-induced downregulation of eNOS activity and NO production. We found that metformin administration significantly increased high salt-induced inhibition of eNOS phosphorylation (Figures 6(a) and 6(b)). Furthermore, high salt-induced inhibition of NO production could also be prevented by metformin (Figures 6(c) and 6(d)).

## 4. Discussion

This study provides evidence that endothelial ENaC is regulated by AMPK and that this regulation may play an important role in dietary salt-induced endothelial dysfunction. The major findings include the following: (1) high salt significantly elevated ENaC expression and activity in endothelial cells; (2) high salt-induced reduction of eNOS activity and NO level were prevented by the specific ENaC blocker, amiloride; (3) AMPK activity was reduced in high salt-treated endothelial cells and metformin, an AMPK activator, significantly reversed high salt-induced elevation of ENaC expression and activity; and (4) activation of AMPK also prevented high salt-induced reduction of eNOS activity and NO level in endothelial cells.

Dietary salt loading in rats is known to result in increase of arterial blood pressure and impairment of endothelium-dependent vascular relaxation. Sodium in the plasma has been suggested to play a primary role in controlling blood pressure because a small increase in plasma sodium level (1–3 mM) was found in individuals with hypertension [23, 24]. Moreover, an acute increase in plasma sodium concentration observed in people on high salt diet has been proposed to alter the mechanical properties of the vascular endothelium [5, 24]. The sodium-selective ion channel, ENaC, is expressed on the surface of endothelial cells; therefore, it could act as a functional link between the plasma and the endothelial cells.

It has been reported that the elevation of plasma Na^+^ concentration stimulates the membrane insertion of *α*-subunit of ENaC in human endothelial cells [16, 25]. Pérez et al. [17] found that the inhibition of endothelial ENaC activates eNOS and increases NO production in mesenteric arteries. In this study, our results showed that high sodium concentration significantly elevates ENaC abundance and activity and reduces eNOS activity and NO level. Here, we showed, for the first time, that *α*-, *β*-, and *γ*-subunits of ENaC are expressed in cultured HUVECs, and high sodium concentration upregulated both the protein level and the channel activity of ENaC. Moreover, the protein abundance of *α*-, *β*-, and *γ*-ENaC was significantly increased by high sodium treatment. Whereas, Wang et al. [15] reported that *α*-subunit of ENaC, but not *β*- and *γ*-subunits, was expressed in cultured endothelial cells by PCR. We speculate that the reason for this discrepancy might be due to the experimental conditions or the difference in antibody preparation. Moreover, our earlier results have also shown that high salt diet inhibits ENaC and leads to the enhancement of acetylcholine-induced relaxation of the vasculature in SD rats, which might be a feedback inhibition of the development of salt-sensitive hypertension [26]. However, high salt diet significantly increased the expression and activity of ENaC and induced hypertension in salt-sensitive rats (our unpublished data). Together, we suggest that high salt challenge upregulates ENaC and leads to endothelial dysfunction, which might play an important role in the development of salt-sensitive hypertension.

AMPK regulates ENaC activity in oocytes, polarized kidney cells, and lung epithelial cells [19, 20, 22]. In this study, we examined the effects of AMPK activation by metformin on ENaC abundance and activity in HUVECs. We first examined the effect of high sodium treatment on activation of AMPK, measured by phosphorylated AMPK*α* appearance. Our data show that phosphorylated AMPK levels in HUVECs decreased 24 h after exposure to additional 20 mM NaCl and that ENaC blockade had no effect on high sodium-induced inhibition of AMPK activation in these cells. The results obtained in kidney regarding the effects of high salt on AMPK activity are controversial. It appeared that, in rat kidney, high salt diet activated AMPK, whereas low salt diet led to inhibition of AMPK activity. Interestingly, both low and high salt media transiently activated AMPK in the cultured macula densa cell line MMDD1, an effect due to changes in osmolarity [27]. In contrast, another study suggests that renal expression of activated AMPK was dramatically decreased in rat fed with high salt intake [28]. These conflicting results suggest that the effects of high salt on AMPK activity may depend upon experimental model and cell types. Nevertheless, we suggest that high salt attenuates AMPK activity in HUVECs.

Accordingly, we examined the effects of metformin on ENaC abundance and activity and found that metformin markedly inhibited the all three subunits of ENaC protein expression and reduced ENaC *P*_*O*_ in HUVECs. These results demonstrate that activation of AMPK abrogates the activated effect of high sodium treatment on ENaC current and expression of *α*-, *β*-, and *γ*-subunits. Since AMPK is a sensor of the “cellular fuel,” which responds to changes in cellular ATP, therefore, AMPK regulation of ENaC might provide a mechanism to adapt to high sodium concentration and/or metabolic stress. There are several lines of evidence to suggest that AMPK inhibits ENaC through functional regulation of the ubiquitin ligase Nedd4-2 [19, 29]. Nedd4-2 interacts with *β*- and *γ*-subunits of ENaC at their C-terminal tails, thereby contributing to the reduction of ENaC cell surface expression [30, 31]. Recent work has also suggested that Nedd4-2 activation may affect opening probability in addition to an effect on cell surface expression of ENaC [32, 33]. Therefore, we speculated that the protective effects of AMPK activation by metformin on ENaC could be mediated by Nedd4-2. However, there are numerous possible mechanisms by which ENaC regulation may be linked to AMPK. Investigating intermediate pathways and underlying mechanisms involved are important goals for future studies.

## 5. Conclusions

Our study suggests that endothelial ENaC is stimulated by high concentration of salt and negatively modulates eNOS in response to high salt treatment. Blocking ENaC in endothelial cells increases eNOS activity and NO production. High salt stimuli-induced enhancement of ENaC expression and activity in HUVECs was downregulated by AMPK. Therefore, AMPK might act directly in the endothelium by inhibiting ENaC expression and activity, thereby contributing to endothelial protection in response to high salt challenge.

## Abbreviations

ENaC: Epithelial sodium channel
AMPK: AMP-activated protein kinase
eNOS: Endothelial nitric oxide synthase
NO: Nitric oxide
HUVEC: Human umbilical vein endothelial cells
*P*_*O*_: Open probability.
